# Treatment with Commonly Used Antiretroviral Drugs Induces a Type I/III Interferon Signature in the Gut in the Absence of HIV Infection

**DOI:** 10.1016/j.xcrm.2020.100096

**Published:** 2020-09-22

**Authors:** Sean M. Hughes, Claire N. Levy, Fernanda L. Calienes, Joanne D. Stekler, Urvashi Pandey, Lucia Vojtech, Alicia R. Berard, Kenzie Birse, Laura Noël-Romas, Brian Richardson, Jackelyn B. Golden, Michael Cartwright, Ann C. Collier, Claire E. Stevens, Marcel E. Curlin, Timothy H. Holtz, Nelly Mugo, Elizabeth Irungu, Elly Katabira, Timothy Muwonge, Javier R. Lama, Jared M. Baeten, Adam Burgener, Jairam R. Lingappa, M. Juliana McElrath, Romel Mackelprang, Ian McGowan, Ross D. Cranston, Mark J. Cameron, Florian Hladik

**Affiliations:** 1 Department of Obstetrics and Gynecology, University of Washington, Seattle, WA, USA; 2 Vaccine and Infectious Disease Division, Fred Hutchinson Cancer Research Center, Seattle, WA, USA; 3 Department of Epidemiology, University of Washington, Seattle, WA, USA; 4 Departments of Obstetrics & Gynecology and Medical Microbiology, University of Manitoba, Winnipeg, MB, Canada; 5 Center for Global Health and Diseases, Case Western Reserve University, Cleveland, OH, USA; 6 Department of Population and Quantitative Health Sciences, Case Western Reserve University, Cleveland, OH, USA; 7 Division of HIV/AIDS Prevention, Centers for Disease Control and Prevention, Atlanta, GA, USA; 8 Thailand Ministry of Public Health-US Centers for Disease Control and Prevention Collaboration, Nonthaburi, Thailand; 9 Department of Medicine, Division of Infectious Diseases, Oregon Health and Sciences University, Portland, OR, USA; 10 Partners in Health Research and Development, Kenya Medical Research Institute, Thika, Kenya; 11 Center for Clinical Research (CCR), Kenya Medical Research Institute (KEMRI), Nairobi, Kenya; 12 Infectious Disease Institute, Makerere University, Kampala, Uganda; 13 Asociación Civil Impacta Salud y Educación, Lima, Peru; 14 Unit of Infectious Diseases, Department of Medicine Solna, Centre for Molecular Medicine, Karolinska Institute, Karolinska University Hospital, Stockholm, Sweden; 15 Department of Pediatrics, University of Washington, Seattle, WA, USA; 16 Department of Medicine, University of Washington School of Medicine, Seattle, WA, USA; 17 Department of Laboratory Medicine, University of Washington, Seattle, WA, USA; 18 Department of Global Health, University of Washington, Seattle, WA, USA; 19 Orion Biotechnology, Ottawa, ON, Canada; 20 Department of Medicine, University of Pittsburgh School of Medicine, Pittsburgh, PA, USA; 21 Department of Infectious Diseases and Dermatology, University of Barcelona, Barcelona, Spain

**Keywords:** HIV, HIV cure, antiretroviral treatment, ART, tenofovir, interferon, chronic immune activation, gut, ISG15

## Abstract

Tenofovir disoproxil fumarate (TDF) and emtricitabine (FTC) are used for HIV treatment and prevention. Previously, we found that topical rectal tenofovir gel caused immunological changes in the mucosa. Here, we assess the effect of oral TDF/FTC in three HIV pre-exposure prophylaxis trials, two with gastrointestinal and one with cervicovaginal biopsies. TDF/FTC induces type I/III interferon-related (IFN I/III) genes in the gastrointestinal tract, but not blood, with strong correlations between the two independent rectal biopsy groups (Spearman r = 0.91) and between the rectum and duodenum (r = 0.81). Gene set testing also indicates stimulation of the type I/III pathways in the ectocervix and of cellular proliferation in the duodenum. mRNA sequencing, digital droplet PCR, proteomics, and immunofluorescence confirm IFN I/III pathway stimulation in the gastrointestinal tract. Thus, oral TDF/FTC stimulates an IFN I/III signature throughout the gut, which could increase antiviral efficacy but also cause chronic immune activation in HIV prevention and treatment settings.

## Highlights

Tenofovir (TDF) and emtricitabine (FTC) are used for HIV treatment and prevention
TDF/FTC induce a type I/III interferon-associated signature throughout the gut
IFN I/III induction is confirmed in independent clinical cohorts and 5 assay types
IFN I/III induction may contribute to anti-HIV efficacy and chronic immune activation

## Graphical Abstract

**Figure:**
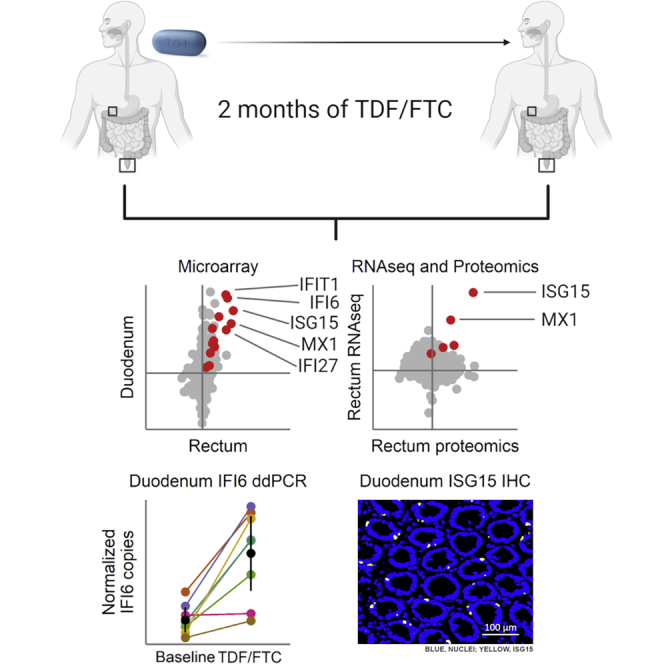

## Teaser

Many people take the antiretroviral drugs tenofovir and emtricitabine to prevent and treat HIV infection. Hughes et al. show that these medications induce genes and proteins associated with type I/III interferon pathways in the gut. This effect may contribute to HIV inhibition but could also cause chronic immune activation.

## Introduction

Despite highly active antiretroviral treatment (ART), persons living with HIV (PLWH) are more likely than HIV-uninfected individuals to experience non-AIDS-related morbidities, such as non-AIDS-defining cancers, cardiovascular disease, osteoporosis, frailty, and other conditions associated with aging.^1^ This increased morbidity appears to be associated, at least in part, with low-level immune activation persisting even in the face of complete suppression of plasma viremia.^2^ The extent to which ART itself may contribute to this chronic immune activation has been a nagging concern without adequate investigation.

The two nucleoside/nucleotide reverse transcriptase inhibitors (NRTIs) tenofovir disoproxil fumarate (TDF) and emtricitabine (FTC) are common components of ART, which must be taken lifelong to prevent clinical progression to AIDS. Tenofovir and emtricitabine in a single combination pill also compose the only licensed oral pre-exposure prophylaxis (PrEP) intervention for uninfected persons at risk of HIV infection.^3^^,^^4^ While tenofovir and emtricitabine are generally well tolerated and considered safe, their effects specifically on the immune system have been little studied. In particular, no studies have evaluated their contribution to immune dysregulation in the gastrointestinal (GI) tract. This is surprising, given that the GI tract harbors the largest HIV burden on and off ART^5^ and is a major source of immune dysfunction in PLWH.^2^ Moreover, two trials of tenofovir application directly to the female genital tract or the rectum as topical PrEP demonstrated a pro-inflammatory effect on the mucosa.^6^^,^^7^ Analogous to these studies of topical PrEP, oral PrEP offers a unique opportunity to define the immunological effects of tenofovir-emtricitabine without interference by HIV infection, HIV-associated comorbidities, or the additional drugs that PLWH must take. Because the oral use of tenofovir and emtricitabine is more common than the topical use of tenofovir by orders of magnitude, lacking information on its mucosal impact in fact constitutes a startling knowledge gap.

To fill this gap, we received and tested blood and mucosal specimens from three human HIV PrEP studies of oral tenofovir-emtricitabine or tenofovir alone, two pre-licensure (NCT00557245 and NCT01687218)^8^^,^^9^ and one post-licensure (NCT02621242). Participants were HIV^−^, generally healthy, and took no additional antiretroviral medications such as integrase or protease inhibitors, allowing us to evaluate the drugs’ effects with minimal confounders. We used transcriptomics and proteomics for broad discovery and confirmed results by focused PCR assays and immunofluorescence staining.

## Results

We measured the effect of oral TDF and FTC, or oral TDF alone, on the GI tract, the female reproductive tract, and blood from three clinical trials of oral PrEP in HIV-uninfected individuals: the Microbicide Trials Network trial 017^9^ (MTN-017); ACTU-3500 (first reported here, see Method Details); and the Genital Mucosal Substudy (GMS)^10^ of the Partners PrEP Study.^8^ We analyzed gene expression by microarray, RNA sequencing (RNA-seq), and digital droplet PCR (ddPCR), and protein expression by mass spectrometry and microscopy. Paired GI biopsies were obtained before and during treatment from two studies: MTN-017 (rectal biopsies) and ACTU-3500 (rectal and duodenal biopsies). Paired female reproductive tract biopsies were obtained during and after treatment from one GMS cohort (GMS A, vaginal and ectocervical biopsies). Paired blood was used from ACTU-3500 and GMS A. Finally, blood was also obtained from a second GMS cohort to compare treatment to placebo (GMS B). Samples and assays are described in Table 1 and in Method Details. RNA quality was assessed for all samples with the RNA integrity number (Table S1).

**Table 1:** Characteristics of Studies and Cohorts from Which Samples Were Obtained

Study	Cohort	Gender	Control	Treatment	Drug	Sample	No.	Assays
NCT01687218^9^	MTN-017	men (34), trans women (2)	pre-treatment, paired	2 months	TDF/FTC	rectum	36 pairs	microarray, RNA-seq, ddPCR, Proteomics
NCT02621242	ACTU-3500	men	pre-treatment, paired	2 months	TDF/FTC	duodenum	8 pairs	microarray, ddPCR, Microscopy
					TDF/FTC	rectum	8 pairs	microarray, ddPCR, Microscopy
					TDF/FTC	whole blood	8 pairs	microarray, ddPCR
					TDF/FTC	PBMC	8 pairs	microarray, ddPCR
NCT00557245^8^^,^^10^	GMS A	women	2 months after treatment cessation, paired	24–36 months	TDF/FTC	vagina	3 pairs	microarray
					TDF	vagina	12 pairs	microarray, ddPCR
					TDF	ectocervix	9 pairs	microarray, ddPCR
					TDF	PBMC	10 pairs	microarray, ddPCR
	GMS B	women	placebo, unpaired	24–36 months	TDF	PBMC	36 drug, 20 placebo	microarray
					TDF/FTC	PBMC	26 drug, 20 placebo	microarray

ACTU-3500, AIDS Clinical Trial Unit Study 3500; FTC, emtricitabine; GMS, Genital Mucosal Substudy of the Partners PrEP study; MTN-017, Microbicide Trials Network study 017; PBMC, peripheral blood mononuclear cells; TDF, tenofovir disoproxil fumarate.

### Differentially Expressed Genes

Gene expression was measured with microarrays, comparing no treatment and treatment with oral TDF or TDF/FTC, paired within individuals for studies with multiple samples per participant (MTN-017, ACTU-3500, and GMS A). Differential expression was defined by a false discovery rate (FDR)-adjusted p < 0.05, with upregulation meaning higher expression during treatment. Differential expression was quantified with log2-fold changes. Differentially expressed genes were found in two studies: MTN-017 rectal samples (13 genes up, n = 36 pairs of samples) and ACTU-3500 duodenal samples (116 genes up and 135 genes down, n = 8 pairs of samples) (Table 2). No differentially expressed genes were found in any of the other study arms by our chosen FDR-adjusted p value threshold of 0.05. Gene lists for every study arm and sample type are found in Data S1.

**Table 2:** Differentially Expressed Genes

Study	Drug	Sample	Genes Up	Genes Down	Total Genes	Off Treatment n	On Treatment n
MTN-017	TDF/FTC	rectum	13	0	21,683	36	36
ACTU-3500	TDF/FTC	duodenum	116	135	16,321	8	8
ACTU-3500	TDF/FTC	rectum	0	0	16,399	8	8
ACTU-3500	TDF/FTC	whole blood	0	0	13,922	8	8
ACTU-3500	TDF/FTC	PBMC	0	0	14,937	8	8
GMS A	TDF/FTC	vagina	0	0	18,397	3	3
GMS A	TDF	vagina	0	0	18,397	12	12
GMS A	TDF	ectocervix	0	0	17,578	9	9
GMS A	TDF	PBMC	0	0	16,553	10	10
GMS B	TDF	PBMC	0	0	20,121	20	36
GMS B	TDF/FTC	PBMC	0	0	20,121	20	26

Differentially expressed genes are defined by an FDR-adjusted p < 0.05, with up indicating higher expression during drug treatment. All of the analyses were paired within individuals, except for GMS B, in which samples from treated individuals were compared to samples from individuals receiving placebo.

All 13 genes differentially expressed in the rectum in MTN-017 were expressed more highly during treatment with TDF/FTC. As shown in Table 3, seven of these 13 genes are members of the Gene Ontology (GO) biological process “type I interferon (IFN) signaling pathway” (GO: 0060337): IFN-α-inducible protein 27 (IFI27), IFI6, IFIT1, IFN-stimulating gene 15 (ISG15), radical S-adenosyl methionine domain containing 2 (RSAD2), MX1, and 2′-5′-oligoadenylate synthetase 1 (OAS1). Four of the other 6 have been reported in the literature to be induced by type I IFN: DDX60,^11^^,^^12^ sterile α motif domain containing 9 (SAMD9),^11^ IFI27L1,^13^ and HECT and RLD domain containing E3 ubiquitin protein ligase family member 6 (HERC6).^11^ Thus, only 2 of the 13 genes (CCDC77 and the pseudogene MROH3P) have no reported roles related to type I IFN. GO overrepresentation analysis of these 13 genes revealed that they were highly overrepresented in biological processes related to type I IFN and response to virus (Data S1). These induced genes are also involved in type III IFN pathways, which play important roles in viral responses at mucosal surfaces, including the rectum.^14^ Neither type I IFNs (IFN-α and -β) nor type III IFNs (IFN-λ1–4) were detectable in our data, so it is unknown which IFN type stimulated these expression changes. Therefore, we refer to these genes as being associated with IFN-I/III pathways.

**Table 3:** Genes Differentially Expressed (Higher during Daily Oral TDF/FTC Use) in Rectal Biopsies in MTN-017

Entrez ID	Gene	Gene Name	Link to Type I/III IFN	Fold Change (log2)	FDR
9636	ISG15	ISG15 ubiquitin-like modifier	gene set	0.98	2.49E−3
4599	MX1	MX dynamin like GTPase 1	gene set	0.92	3.60E−3
2537	IFI6	IFN α-inducible protein 6	gene set	0.80	2.27E−4
3429	IFI27	IFN α-inducible protein 27	gene set	0.76	3.54E−7
3434	IFIT1	IFN-induced protein with tetratricopeptide repeats 1	gene set	0.75	2.49E−3
91543	RSAD2	radical S-adenosyl methionine domain containing 2	gene set	0.53	3.14E−3
55601	DDX60	DExD/H-box helicase 60	literature^11^^,^^12^	0.39	2.49E−3
54809	SAMD9	sterile α motif domain containing 9	literature^11^	0.35	0.012
4938	OAS1	2′-5′-oligoadenylate synthetase 1	gene set	0.32	0.016
55008	HERC6	HECT and RLD domain containing E3 ubiquitin protein ligase family member 6	literature^11^	0.31	0.049
122509	IFI27L1	IFN α-inducible protein 27-like 1	literature^13^	0.25	2.27E−4
647215	MROH3P	maestro heat-like repeat family member 3, pseudogene	none reported	0.22	0.021
84318	CCDC77	coiled-coil domain containing 77	none reported	0.12	0.048

Gene set, membership in GO: 0060337; literature, indicates that a link to type I/III interferon has been reported in the indicated articles. IFN, interferon.

In the duodenum in ACTU-3500, the top overrepresented biological processes for downregulated genes were related to cellular metabolism, and for upregulated genes they were related to a variety of biological processes, including RNA splicing and phospholipid transport (Data S1).

### Correlation of Gene Expression across Study Arms

**Figure 1:**
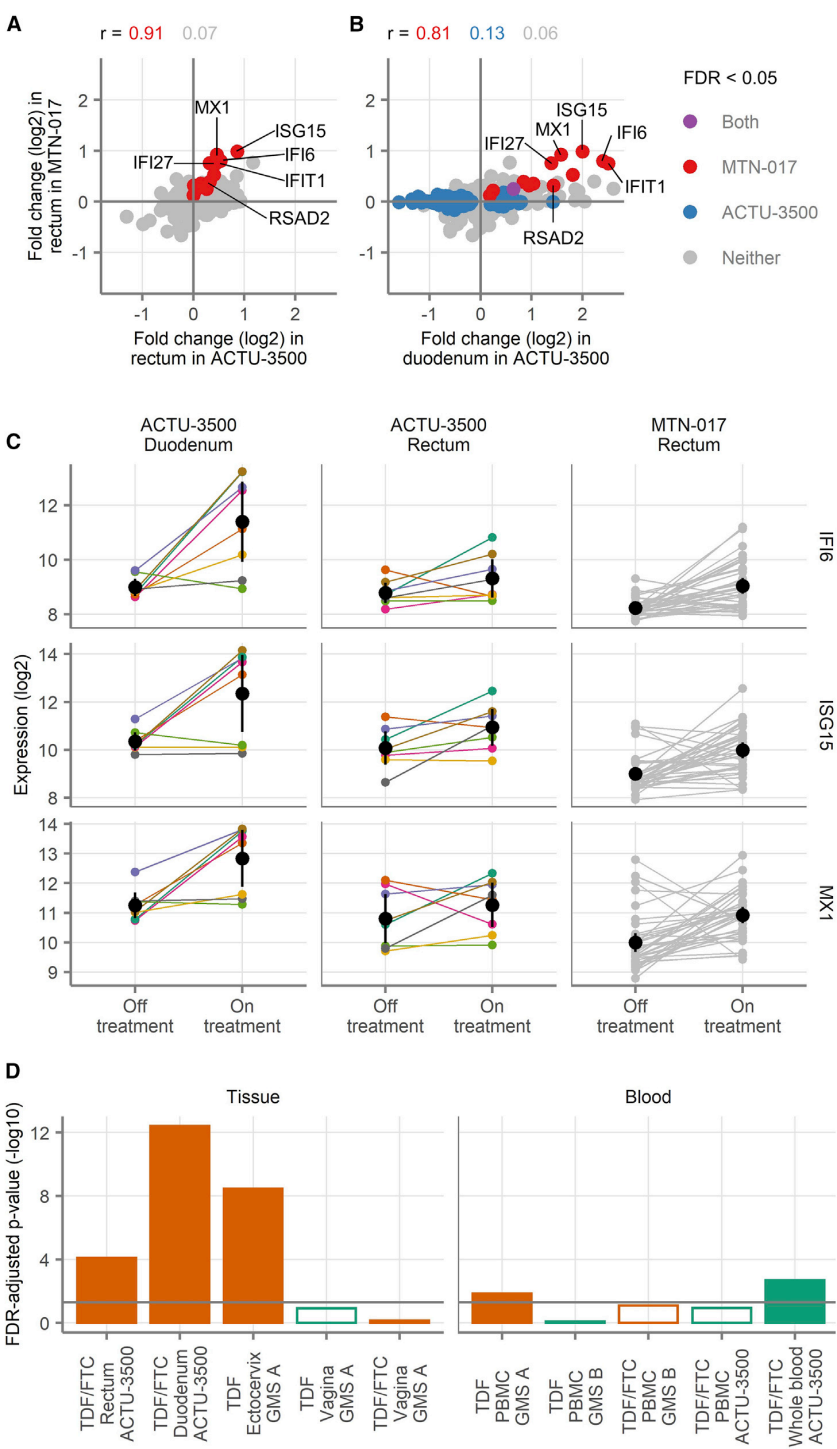
Gene Expression across Study Arms by Microarray Analysis (A and B) Fold changes of all genes detected in the rectal samples in MTN-017 compared to the rectal samples from ACTU-3500 (A) and the duodenal samples from ACTU-3500 (B). The colors indicate genes with FDR-adjusted p < 0.05 in MTN-017 (red), ACTU-3500 (blue), both (purple), or neither (gray). Spearman correlation coefficients for the genes falling into each subset are shown. (C) Expression levels of IFI6, ISG15, and MX1 in individual samples. Small points indicate measurements from a single biopsy, with lines connecting the matching observation from the same donor. For ACTU-3500, the color of the lines and symbols indicate the participant and are consistent across the panels. The black symbols and vertical lines show the means and 95% confidence intervals of the mean. (D) Gene set testing of a custom gene set composed of differentially expressed genes from the rectum in MTN-017 performed in the other study arms in the mucosa (left) and blood (right), comparing the expression of genes in the set to all of the other detected genes within each study arm. The bars indicate the result of a test against the study labeled on the x axis. The filled bars indicate an FDR-adjusted p < 0.05 and open symbols the opposite, with the bar height showing the −log10 of the FDR-adjusted p value. The colors indicate the direction of change, with orange indicating more expression during product use and green indicating the opposite. The horizontal gray line shows an FDR-adjusted p = 0.05. The sample sizes are shown in Table 1. Each sample was run in singlicate by microarray.

The log2-fold changes of the 13 differentially expressed genes from the rectum in MTN-017 strongly correlated with the fold changes of these same genes in the rectum in ACTU-3500 (Figure 1A) (Spearman correlation coefficient r = 0.91, as compared to 0.07 for all other genes). None of these genes had adjusted p < 0.05 in the rectum in ACTU-3500 (two had unadjusted p < 0.05), possibly due to the much smaller sample size.

Similarly, there was a strong correlation between the fold changes of the 13 differentially expressed genes in the rectum from MTN-017 with the fold changes of the same genes in the duodenum in ACTU-3500 (Figure 1B) (Spearman correlation coefficient r = 0.81, as compared to 0.13 for all of the genes that were differentially expressed in the duodenum, and 0.06 for all other genes). Only 1 of these genes had an adjusted p < 0.05 in the duodenum in ACTU-3500 (although all 13 had unadjusted p < 0.05), possibly due to the much smaller sample size.

These results show that oral TDF or TDF/FTC affect the expression of relatively few genes. In particular, we did not find evidence of differential gene expression in the blood. We did detect differentially expressed genes in the rectum and the duodenum. The 13 genes that were differentially expressed in the rectum in MTN-017 were strongly correlated in the duodenum and rectum in ACTU-3500. Microarray gene expression levels for the 3 genes with the highest fold changes (IFI6, ISG15, and MX1) are depicted for all of the individual samples in Figure 1C.

We additionally performed gene set testing across each of the other study arms by using these 13 genes as a custom gene set, comparing their expression with the expression of genes outside the set. This custom 13-gene set derived from the rectum in MTN-017 was strongly enriched in the rectum and duodenum in ACTU-3500 (FDR = 8E−5 and 4E−13, Figure 1D, and complete gene set testing results in Data S1), as well as in the ectocervix. This suggests that those 13 genes, 11 of which are known to be associated with type I/III IFN pathways, may represent an underlying biological process that is affected by TDF/FTC in the GI tract.

### Gene Set Testing of Hallmark Gene Sets

**Figure 2:**
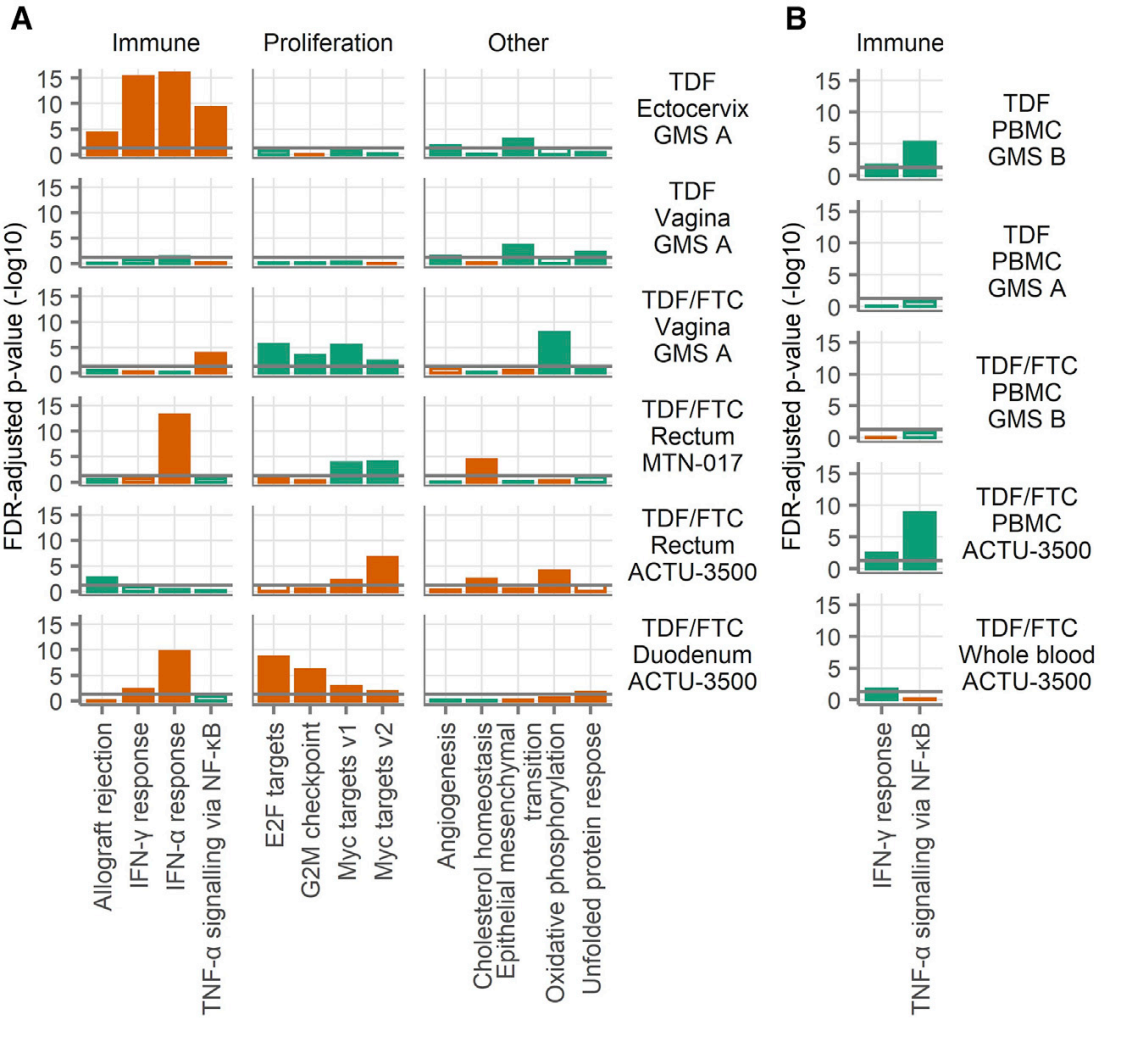
Hallmark Gene Sets (A and B) Gene set testing was performed on the Hallmark gene sets in the mucosa (A) and the blood (B). To reduce the number of gene sets displayed and focus on those gene sets that were affected in multiple studies, we show only those gene sets that had an FDR-adjusted p < 0.05 in at least two of the mucosa (A) or blood (B) study arms. The bars indicate the result of a gene set test for the gene set shown on the x axis tested against the study shown at right. The filled bars indicate an FDR-adjusted p < 0.05 and open bars the opposite, with the bar length proportional to the −log10 of the FDR-adjusted p value. The colors indicate the direction of change, with orange indicating more expression during product use and green indicating less expression. The horizontal gray lines show an FDR-adjusted p = 0.05. The gene sets are grouped into categories as labeled at top. The sample sizes are shown in Table 1. Each sample was run in singlicate by microarray.

To assess higher-level biological effects, we performed gene set testing on the 50 Hallmark gene sets^15^ within each study arm and specimen type, comparing the expression of genes in each set to the expression of genes not in the set (i.e., all other detected genes). Each gene set comprises genes that are involved in a biological state or process. To reduce the number of gene sets displayed and focus on those gene sets that were affected in multiple studies, we show the gene sets that had adjusted p < 0.05 in at least two study arms for tissue and blood in Figures 2A and 2B. Complete gene set testing results are provided in Data S1. For tissue (Figure 2A), 13 gene sets had adjusted p < 0.05 in at least two study arms. Of these gene sets, four were related to immunity (allograft rejection, IFN-α and -γ responses, and tumor necrosis factor α [TNF-α] signaling via nuclear factor κB [NF-κB]), and four were related to cell proliferation (E2F targets, G2M checkpoint, and two MYC target gene sets). In the mucosa, the immune-related gene sets tended to be elevated during product use, with the IFN-α response being the strongest, not only in the duodenum and rectum but also in the ectocervix. Only two gene sets had adjusted p < 0.05 in at least two study arms for blood samples (Figure 2B). Both gene sets were immune related (complement, IFN-γ response, and TNF-α signaling via NF-κB). In both cases, these gene sets were lower during product use. Thus, TDF/FTC seemed to induce IFN-α responses in the GI tract and ectocervix but had a somewhat dampening effect on inflammatory responses in the blood.

### Corroboration of Microarray Data by RNA-Seq, Mass Spectrometry, and ddPCR

**Figure 3:**
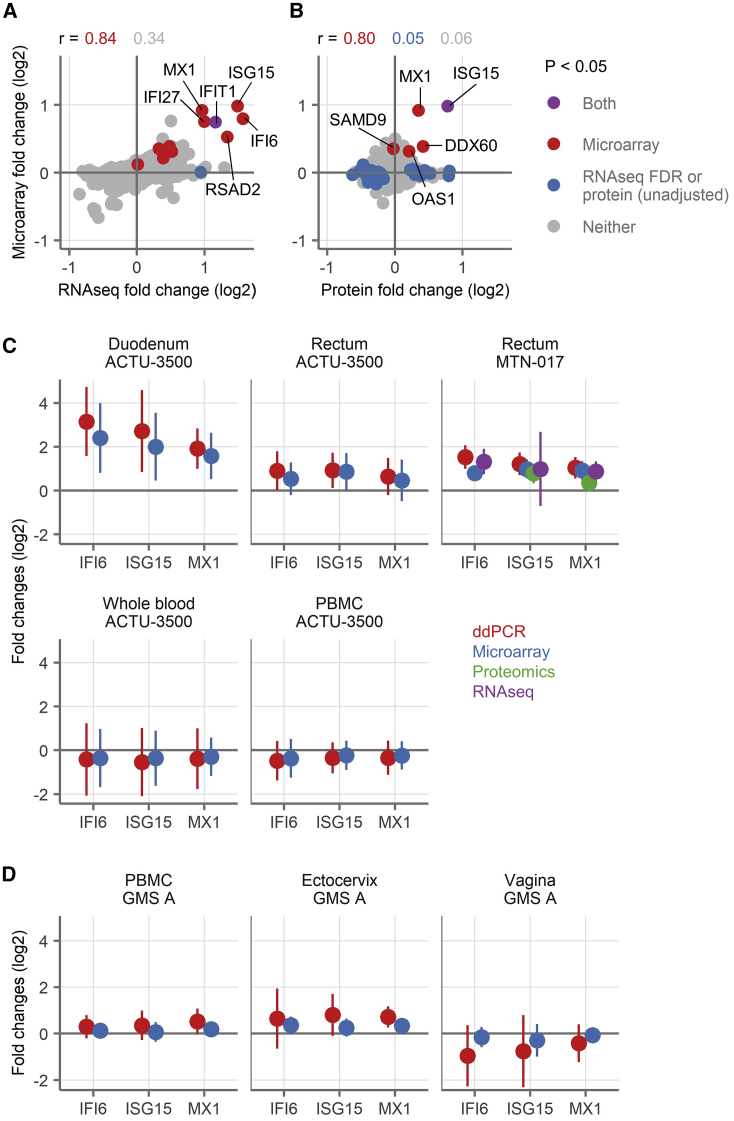
Comparison of Gene Expression Changes Measured by ddPCR, RNA-Seq, Proteomics, and Microarray (A and B) Correlation of fold changes of genes as detected by microarray (y axis) with genes detected by RNA-seq (A) or proteins detected by proteomics (B) from the rectal samples from MTN-017. The colors indicate genes with FDR-adjusted p < 0.05 in microarray transcripts (red), RNA-seq genes (blue, by FDR, A) or proteins (blue, unadjusted p value, B), both (purple, FDR for microarray and RNA-seq, unadjusted p value for protein), or neither (gray). Spearman correlation coefficients for the genes falling into each subset are shown. Selected genes are labeled. (C and D) Fold changes in gene expression of three genes (IFI6, ISG15, or MX1) as detected by ddPCR (red), microarray (blue), proteomics (green), and RNA-seq (purple) after treatment with TDF/FTC (C) or TDF alone (D). The symbols show the mean across all of the participants and vertical lines show the 95% confidence intervals of the mean. ISG15, ISG15 ubiquitin-like modifier; MX1, MX dynamin-like GTPase 1. A positive fold change means higher expression during treatment, and a negative fold change means higher expression off treatment. For ddPCR, the expression of each gene of these three genes was normalized to the expression of ubiquitin C (UBC), which was chosen as reference due to the stability of its expression across tissues and treatments in the microarray data. The sample sizes are shown in Table 1. Each sample was run in singlicate by RNA-seq, proteomics, and microarray, while samples were run in duplicate technical replicates by ddPCR.

In addition to microarrays, we analyzed the RNA from MTN-017 participants by RNA-seq. Two genes were differentially expressed (FDR-adjusted p < 0.05) in the RNA-seq data, both higher during TDF/FTC treatment than at baseline: IFIT1, which was also differentially expressed by microarray, and SLC6A20, which was not. Fold expression changes of the 13 genes identified as significantly upregulated in the microarrays correlated strongly with their fold expression changes by RNA-seq (Spearman correlation 0.84 as compared to 0.34 for all other genes Figure 3A). By virtue of having both microarray and RNA-seq data on the same samples, we were able to look at genes that had similar fold changes by using both methodologies. We looked at genes that had log2-fold changes <−0.25 or >0.25 with both assays. Only 12 genes fell into the downregulated group and none had adjusted p < 0.05 by either assay, but eight were metallothioneins (MT1A, 1E, 1F, 1G, 1H, 1M, 1X, and 2A), which bind to heavy metal ions. There were 28 genes with fold changes >0.25 by both microarray and RNA-seq, including all of the differentially expressed genes from the microarray, except for the two non-type I/III IFN-related genes (i.e., 11 of 13 microarray genes). Many of the additional genes were also related to type I/III IFN signaling: IRF7, IRF9, OAS2, OAS3, IFITM1, and IFI44L, for example. An overrepresentation analysis of these 28 genes (with or without the 11 differentially expressed genes from the microarray) again yielded many GO biological processes related to type I/III IFN signaling.

We also analyzed rectal biopsies from MTN-017 by mass spectrometry for protein identification. The biopsies were run in two batches, with the samples from US participants in one batch and samples from Thai participants in the other. In both cases, no proteins were detected as differentially expressed after adjustment for multiple comparisons (complete list of protein fold changes in Data S1). None of the proteins from the 13 genes of interest was consistently detected in the US participants, due to the low abundance of these proteins. We therefore could not analyze the US batch for these proteins. However, five of these proteins (ISG15, MX1, OAS1, DDX60, and SAMD9) were detected in all of the Thai samples. All but one (SAMD9) had positive fold changes and there was a strong correlation between the fold changes detected by microarray and mass spectrometry (Spearman 0.80; Figure 3B).

Finally, we used ddPCR to measure the expression of three selected IFN-I/III genes (IFI6, ISG15, and MX1). The fold changes for ddPCR and microarray are shown in Figures 3C and 3D. By ddPCR, linear scale fold changes for these three genes ranged from 3.77 to 8.89 in the duodenum and 1.56 to 2.88 in the rectum, while they were little changed in the blood, ectocervix, and vagina. The changes measured by ddPCR were always in the same direction as those by microarray, and the magnitudes tended to be larger by ddPCR. The participant-level fold changes calculated from ddPCR measurements correlated well with the microarray data (Pearson correlation 0.93 for MX1, 0.92 for ISG15, and 0.90 for IFI6). Taking each gene separately and stratifying by study, sample type, and gene, Pearson correlations ranged from 0.54 to 0.99, with a mean of 0.86 and median of 0.92. Thus, the ddPCR data confirmed that oral TDF/FTC induces genes related to type I/III IFN throughout the gut and mildly in the ectocervix, but not the vagina and blood.

The data obtained from the rectum and the duodenum after TDF/FTC treatment for 2 months, in two different studies (MTN-017 and ACTU-3500), and by several different assays (microarray, RNA-seq, ddPCR, and mass spectrometry-based proteomics) indicate the upregulation of factors related to type I/III IFN signaling in the GI tract.

### Immunofluorescence Microscopy of GI Tissues

**Figure 4:**
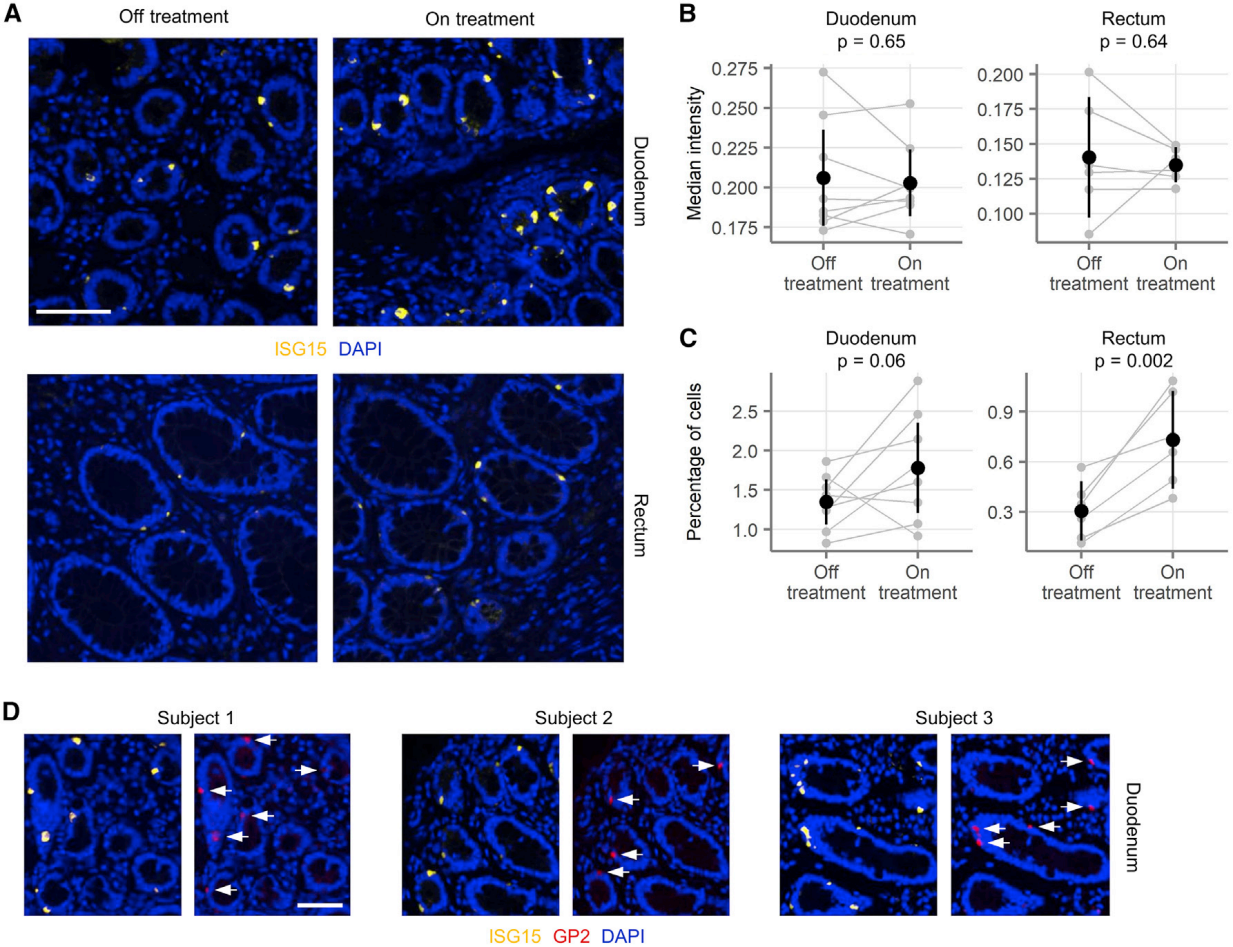
Immunofluorescence Microscopy Staining for ISG15 (A) 20× magnification images of duodenal (top) and rectal (bottom) biopsies, stained for ISG15 (yellow) and DAPI (blue). Biopsies from pre-treatment (left) and at the end of 2 months of treatment (right) are shown. Scale bar, 100 μm. The duodenal biopsies came from one donor and the rectal biopsies came from a second donor. (B) ISG15 intensity on ISG15^+^ cells was measured in paired duodenal (n = 8 donors) and rectal (n = 6) biopsies from ACTU-3500. The median intensity of all of the cells measured is shown. (C) The percentage of ISG15^+^ cells out of all epithelial cells is shown for the same biopsies as in (B). In (B) and (C), gray points indicate measurements from a single biopsy, with gray lines connecting the matching observation from the same donor. The black symbols and vertical lines show the means and 95% confidence intervals of the mean. p values are indicated from one-sided paired t tests. (D) Co-staining of duodenal biopsies from three individuals for ISG15 and glycoprotein 2 (GP2). Anti-GP2 was raised against a peptide component of pancreatic secretory (zymogen) granules and has some cross-reactivity with microfold (M) cells. GP2^+^ cells co-express ISG15 (white arrows), but not all ISG15^+^ cells are GP2^+^. Scale bar, 100 μm.

To further assess the induction of signals associated with type I/III IFN pathways, we stained duodenal and rectal tissue sections from ACTU-3500 for ISG15. Slides were evaluable from 8 pairs of duodenal biopsies and 6 pairs of rectal biopsies (before and during treatment). ISG15 was expressed by few, if any, stromal cells, but some cells within the columnar epithelium stained intensely positive (Figure 4A). Whereas the intensity of ISG15 staining did not change with TDF/FTC use (Figure 4B), the mean percentage of ISG15 bright cells increased in both the rectum (increase of 0.43 percentage points or 2.76-fold, one-sided paired t test p = 0.0023) and the duodenum (0.43 percentage points or 1.37-fold, one-sided paired t test p = 0.06) (Figure 4C). Co-staining of duodenal biopsies from three individuals with ISG15 and glycoprotein 2 (GP2), a cell surface receptor reported to identify microfold (M) cells (a special type of intestinal epithelial cell with heightened immunological activity^16^, ^17^, ^18^), revealed a startling overlap between the two markers (Figure 4D). Thus, TDF/FTC use increased the proportion of cells expressing high levels of ISG15, and at least some of these cells may be M cells.

## Discussion

We found that oral treatment with TDF/FTC or TDF alone had limited global effects on host gene expression, with no differentially expressed genes in the blood or female reproductive tract and tens to hundreds in the GI tract. Notably, however, genes associated with type I/III IFN pathways were consistently induced in the gut, with good agreement between microarray, ddPCR, and RNA-seq. Protein-level data by mass spectrometry and immunohistology of gut sections for ISG15 were in congruence.

The overall limited changes that we saw indicate that TDF/FTC have few off-target effects on host gene expression, suggesting that TDF/FTC treatment is largely benign. In light of the widespread use of TDF/FTC among HIV-infected individuals for treatment and HIV-uninfected individuals for prevention, this is reassuring. Moreover, increased type I/III IFN signaling could signify increased innate immune readiness, enhancing the antiviral preventive or treatment efficacy of TDF/FTC. In fact, induction of a type I/III IFN response signature by TDF/FTC primarily in the GI mucosa could contribute to the greater efficacy of oral PrEP in preventing rectal over vaginal HIV transmission.^19^ Other proposed explanations for this observation include pharmacokinetic differences in TDF levels between vaginal and rectal tissues^20^ and perturbations of TDF metabolism by a dysbiotic vaginal microbiome.^21^

The stimulation of IFN pathways by TDF/FTC could have detrimental effects as well. It is widely known that continued disinhibition of IFN-I/III responses plays an important role in chronic inflammatory diseases.^22^, ^23^, ^24^ Long-term use of TDF/FTC with ongoing stimulation of IFN-I/III pathways could predispose individuals to chronic immune activation, including PLWH. Two recent papers regarding HIV-infected humanized mice showed that blocking IFN-I/III pathways led not only to less immune activation but also to a lower HIV reservoir and delayed HIV rebound following ART interruption.^25^^,^^26^ Oral TDF/FTC in PLWH could therefore paradoxically promote HIV reservoir persistence, especially in the gut, where >99% of the latent reservoir is thought to reside^5^ and where the effect of TDF/FTC is strongest. Thus, selecting ART drugs that do not stimulate signals associated with IFN-I/III pathways may decrease morbidity associated with chronic immune activation and even contribute to HIV cure strategies, which will likely be delivered alongside suppressive ART.^27^ However, we emphasize that these possible consequences of TDF/FTC treatment in PLWH remain speculative because we did not study these drugs specifically in PLWH. Notably, other factors may cause or contribute to persistent immune activation, such as dysregulation of the gut microbiome (reviewed by Dillon et al.^28^), dysfunction of regulatory immune cells,^29^^,^^30^ insults created by HIV infection during the untreated disease phase,^31^ and a number of other possible mechanisms (reviewed by Younas et al.^32^).

In addition to changes to immune-related gene expression in the GI tract, other notable gene expression changes were related to cell proliferation. Specifically, we saw some evidence of increased expression of gene sets related to cell proliferation in the duodenum and reduced expression of these gene sets in the vagina. Proliferation-related gene sets were conflicting in the rectum (two sets up in one study and down in the other). In a prior study of rectal tenofovir 1% gel, we noted the induction of pro-proliferative pathways on both the transcriptomic and proteomic levels.^7^ It is difficult to speculate about the clinical relevance of changes to cell proliferation pathways. Factors associated with cell-cycle regulation are crucial to balance cell proliferation with cell death and for cells to respond to DNA damage. The effect of TDF/FTC on cell-cycle processes in the duodenum may not be surprising, given the increased drug concentrations likely achieved in the upper GI tract. Long-term, high concentration oral administration of TDF to mice has been reported to cause a low incidence of duodenal tumors (Canadian product monograph for Viread) and liver adenomas (US prescribing information for Viread). It is unclear whether our findings with human duodenal biopsies relate in any way to these outcomes in rodents, and no such findings have been reported during human use.

It remains a matter of speculation how TDF/FTC induce a type I/III IFN signature in the gut, but our and another group’s recent data point to some possible mechanisms. First, TDF/FTC increased the relative number of ISG15 bright cells within the columnar epithelium of the gut, but not their ISG15 expression levels (Figure 4), suggesting that the drugs have a proliferation-inducing effect, especially on these cells. Based on the co-expression of GP2, they may be M cells, which are highly active immunological sentinels of the intestinal mucosa.^16^, ^17^, ^18^ The increased numbers of these cells suggest that the increased ISG activity we observed is due to a greater number of ISG-producing cells, rather than more ISGs produced by a constant number of cells. Second, TDF treatment has been reported to increase serum IFN-λ3 levels in patients with hepatitis B or HIV as well as IFN-λ3 secretion from cultured colon cancer cell lines,^33^ indicating a role for type III IFN. These experiments showed that TDF induction of IFN-λ3 production is unique to gut cells and, furthermore, that TDF-induced IFN-λ3-containing supernatants cause the production of MX1 and OAS2. Thus, these experiments provide direct *in vitro* confirmation of our findings that TDF/FTC induce type I/III IFN production specifically in the GI tract. Unfortunately, neither type I nor type III IFNs were detectable in any of our samples, likely due to the inadequate sensitivity of the assays. Lastly, there have been several reports, including our prior finding,^7^^,^^34^^,^^35^ that TDF inhibits interleukin-10 (IL-10) production. It has recently been shown that tenofovir monophosphate, an intracellular metabolite of TDF, strongly binds to Akt, a protein kinase broadly involved in intracellular signaling, including lipopolysaccharide (LPS)-induced stimulation of IL-10 transcription. Binding by tenofovir monophosphate prevents Akt phosphorylation and translocation to the plasma membrane, interrupting a key event in the LPS/IL-10 signaling cascade.^34^ By consequence, TDF treatment reduces anti-inflammatory IL-10 responses to LPS in favor of pro-inflammatory IL-12 responses. These data demonstrate specific intracellular effects of tenofovir, with consequences ranging from disinhibition of cellular proliferation to perturbation of innate cytokine networks. However, more studies will be required to tie these findings together in a unified functional model.

### Limitations of Study

Our study has a few limitations. The first is the use of within-person comparisons between being on and off the drug. Because participants were aware of when they were or were not taking an intervention, behavioral changes or other factors than the drug itself could explain the gene expression changes that we observed. The only study in which placebo was compared to treatment is the GMS B study, in which the samples were peripheral blood mononuclear cells (PBMCs) and no differentially expressed genes were observed. Second, co-existing or new bacterial or viral infections could explain the IFN responses we attributed to the effect of TDF/FTC use. However, it is relatively unlikely for infections to explain these effects, because they would have to occur in a concerted fashion primarily at the end of the study period, during TDF/FTC use, and rarely at baseline, before TDF/FTC initiation. Moreover, the underlying clinical trials were well monitored and included a comprehensive package of HIV prevention counseling and sexually transmitted infection (STI) testing throughout the studies. No increase in STIs or other infections was noted during TDF/FTC use by testing or clinical symptoms. Third, we looked for gene expression changes at the bulk cell levels of tissue, PBMC, and whole blood. Had we looked at specific cell types, we may have seen different results. For example, there is evidence that PrEP alters the composition of immune cells in tissues in the female genital tract and the blood.^35^ We were also limited in our ability to differentiate the effects of FTC from those of TDF, given that only a limited set of participants received TDF alone and none received FTC alone. Finally, and most important, our results are limited to HIV-uninfected people, so it is unclear whether our findings extend to PLWH. With the relatively recent emergence of NRTI-sparing, but equally effective, combination ART (cART) regimens,^36^^,^^37^ it has become possible to conduct a prospective clinical trial comparing immune activation and HIV reservoir decay in PLWH randomized to NRTI-containing versus NRTI-sparing regimens. Our data advocate for such a study.

## STAR★Methods

### Key Resources Table

**Table:** 

REAGENT or RESOURCE	SOURCE	IDENTIFIER
**Antibodies**		

Rabbit polyclonal anti-ISG15	Atlas Antibodies	Cat#HPA004627, RRID: AB_1079152
Rabbit polyclonal anti-GP2	Thermo Fisher	Cat#PA5-42593, RRID: AB_2608499

**Biological Samples**		

Human PBMC from the ACTU-3500 trial	This paper	N/A
Human whole blood from the ACTU-3500 trial	This paper	N/A
Human rectal biopsies from the ACTU-3500 trial	This paper	N/A
Human duodenal biopsies from the ACTU-3500 trial	This paper	N/A
Human PBMC from GMS A	This paper	N/A
Human cervical biopsies from GMS A	This paper	N/A
Human vaginal biopsies from GMS A	This paper	N/A
Human PBMC from GMS B	This paper	N/A
Human rectal biopsies from MTN-017	This paper	N/A

**Critical Commercial Assays**		

HumanHT-12 v4 Expression BeadChips	Illumina	N/A (discontinued)
Ovation PicoSL WTA System V2 kit and Encore BiotinIL kit	NuGEN/TECAN	Cat#3302-12, Cat#4210-48
Illumina TotalPrep RNA Amplification kit	Thermo Fisher	Cat#AMIL1791
qScript cDNA Synthesis Kit	QuantaBio	Cat#95047-025
ddPCR Supermix for Probes (no dUTP)	Bio-Rad	Cat#186-3024
TruSeq Stranded Total RNA with Ribo-Zero Globin kit	Illumina	Cat#20020612

**Deposited Data**		

Microarray data from the ACTU-3500 trial (effect of oral TDF/FTC on gene expression in the blood, duodenum, and rectum)	This paper	GEO: GSE139611
Microarray data from MTN-017 (effect of oral TDF/FTC or topical rectal 1% tenofovir gel on gene expression in the rectum)	This paper	GEO: GSE138723
Microarray data from GMS A (effect of oral TDF/FTC or oral TDF on gene expression in the blood, cervix, and vagina)	This paper	GEO: GSE139655
Microarray data from GMS B (effect of oral TDF/FTC or oral TDF on gene expression in the blood as compared to placebo)	This paper	GEO: GSE139411
RNaseq data from MTN-017 (effect of oral TDF/FTC or topical rectal 1% tenofovir gel on gene expression in the rectum)	This paper	https://doi.org/10.6084/m9.figshare.c.4704827.v2
Mass spectrometry-based proteomics data from MTN-017 (effect of oral TDF/FTC on gene expression in the rectum)	This paper	https://doi.org/10.6084/m9.figshare.c.4704827.v2
ddPCR data from ACTU-3500, MTN-017, and GMS A (effect of oral TDF/FTC or oral TDF on gene expression in the blood, cervix, duodenum, rectum, and vagina)	This paper	https://doi.org/10.6084/m9.figshare.c.4704827.v2

**Oligonucleotides**		

Primers/probes for ddPCR	IDT	Table S2

**Software and Algorithms**		

lumi package	^38^	https://www.bioconductor.org/packages/release/bioc/html/lumi.html
limma package	^39^	https://www.bioconductor.org/packages/release/bioc/html/limma.html
R	CRAN	https://cran.r-project.org/
Tidyverse	CRAN	https://cran.r-project.org/web/packages/tidyverse/index.html

### Resource Availability

#### Lead Contact

Further information and requests for resources and reagents should be directed to and will be fulfilled by the Lead Contact, Florian Hladik (florian@uw.edu).

#### Materials Availability

This study did not generate new unique reagents.

#### Data and Code Availability

The accession numbers for the microarray data reported in this paper are GEO: GSE139611, GSE138723, GSE139655, GSE139411. The original data for all other assays have been deposited on figshare: https://doi.org/10.6084/m9.figshare.c.4704827.v2. All code necessary to reproduce the analyses and figures is provided in Data S2.

### Experimental Model and Subject Details

Only human samples were used in this study. Samples were used from four cohorts from three studies, which are described in Table 1. Two cohorts were used from the Genital Mucosal Substudy (GMS)^10^ of the Partners PrEP Study^8^: paired samples during and after treatment (GMS A) and unpaired placebo versus treatment samples (GMS B). The samples all came from the same parent study, but the two cohorts were processed separately. The Microbicide Trials Network trial 017 (MTN-017)^9^ included oral TDF/FTC as well as topical tenofovir; only the oral TDF/FTC samples are included in this analysis. Rectal biopsies taken after two months of oral TDF/FTC use were compared to baseline samples. The study visits for the samples used here were performed at two sites (Pittsburgh, USA and Bangkok, Thailand), with half of the samples coming from each site. ACTU-3500 followed nine men initiating oral PrEP with TDF/FTC in Seattle, with a baseline visit and a visit after two months of PrEP use. Eight men completed both visits.

Ethics reviews are published in the primary manuscripts for the GMS and MTN-017 studies (listed in Table 1). ACTU-3500 was reviewed through the University of Washington Institutional Review Board, number 49167. Written informed consent was obtained from all participants. Sample sizes varied within each trial depending on drug (TDF/FTC or TDF) and sample type. Complete sample size information is listed in Table 1.

### Method Details

#### Adherence

Tenofovir levels were measured in the serum for the GMS A and GMS B studies as previously described.^57^ Samples from the treatment arm without detectable tenofovir were removed, as was one post-treatment sample, where drug was unexpectedly detected. Tenofovir levels were measured in serum in the MTN-017 study. Adherence was high in the oral arm of this study, with 94% of participants taking the daily pill at least 80% of the time.^58^ Participant report of pill use was largely concordant with serum tenofovir levels, with only 4.4% of serum samples having no detectable tenofovir when participants reported product use. All samples from this study were included. Adherence was determined for the ACTU-3500 study by participant self-report. All participants reported daily use of TDF/FTC throughout the study period.

#### Samples and sample storage

Vaginal, cervical, and rectal biopsies were obtained in GMS A and B and MTN-017 as described in the primary manuscripts.^8^, ^9^, ^10^ Rectal biopsies were obtained in ACTU-3500 by anoscopy using Radial Jaw 4 biopsy forceps (Boston Scientific). Duodenal biopsies were obtained by esophagogastroduodenoscopy under light anesthesia also using Radial Jaw 4 biopsy forceps.

Biopsies were placed into RNALater Stabilization Solution (ThermoFisher Scientific, Waltham, MA, USA) and held at 4°C for 24 h and then frozen at −80°C. PBMC were isolated from whole blood by density gradient centrifugation and then cryopreserved and stored in the vapor phase of a liquid nitrogen freezer. Whole blood was drawn into PAXgene tubes (PreAnalytiX, Hombrechtikon, Switzerland), which were frozen at −20°C for 24 h and then stored at −80°C.

#### RNA extraction and quality control

Biopsies were thawed at room temperature. They were transferred with forceps into 600 μL of Buffer RLT (QIAGEN, Hilden, Germany) and homogenized using a Bio-Gen PRO200 homogenizer (PRO Scientific, Oxford, CT, USA) followed by passing 10 times through a needle and syringe. RNA was extracted using the RNeasy fibrous tissue mini kit (QIAGEN) automated on a QIAcube (QIAGEN). PBMC were thawed, washed by centrifugation, counted, and RNA was extracted using the RNeasy mini kit (QIAGEN) on a QIAcube. PAXgene tubes were thawed and held at room temperature for 3 h with occasional mixing, in order to completely lyse red blood cells. RNA was extracted from PAXgene samples using the PAXgene Blood RNA kit (PreAnalytiX) according to the manufacturer’s instructions. RNA was stored at −80°C until use.

#### Sample quality control

Cell viability was measured prior to RNA extraction for PBMC samples (Guava Viacount, EMD Millipore, Burlington, MA, USA). The quality of all RNA was determined using the RNA integrity number as calculated from the TapeStation R6K assay (Agilent, Santa Clara, CA, USA, Table S1) and the concentration was determined by NanoDrop (ThermoFisher).

#### Microarray labeling and hybridization

For the GMS A and GMS B studies, samples were prepared for microarray using 50 ng of total RNA with the Ovation PicoSL WTA System V2 kit (NuGEN, San Carlos, CA, USA) and labeled with the Encore BiotinIL kit (NuGEN). The Illumina TotalPrep RNA Amplification kit (ThermoFisher) was used to prepare samples for microarray from the ACTU-3500 study (275 ng total RNA as input) and the MTN-017 study (500 ng input), with input sizes chosen based on available RNA.

750 ng of the labeled cDNA (from the NuGEN kits) or cRNA (from the Thermo kit) was hybridized to HumanHT-12 v4 Expression BeadChips (Illumina, San Diego, CA, USA) and scanned by the Fred Hutch Genomics Core facility. Images were converted to expression data using GenomeStudio (Illumina).

#### Reverse transcription and ddPCR of selected genes

We used all the paired samples with enough RNA available to repeat measurements of transcript levels for MX dynamin like GTPase 1 (MX1), ISG15 ubiquitin-like modifier (ISG15), and interferon-α inducible protein 6 (IFI6) by ddPCR assay. As the reference gene, we used Ubiquitin C (UBC), selected based on a comparison of commonly used reference genes in the microarray data. Among those genes, UBC was expressed in all sample types and the average fold change (across different sample types and studies) was close to 0 and the standard deviation was small. The samples used for ddPCR were the same as used for microarray, except that the GMS B samples were not used (because they were unpaired), the number of sample pairs was reduced by one each for the vaginal and ectocervical samples from the TDF arm of the GMS A study because of insufficient RNA remaining from those samples. The vaginal samples from the TDF/FTC arm of the GMS A study were not tested by ddPCR because the sample size was so low (only three pairs of samples).

Reverse transcription was performed using 100 ng of RNA per sample in a 20 μL reaction mixture using qScript cDNA Synthesis Kit (QuantaBio, Beverly, MA, USA) according to the manufacturer’s instructions. The incubation conditions were 22°C for 5 minutes, 42°C for 30 min, and then 85°C for 5 min. After reverse transcription, the samples were diluted to 100 μL with water and 5 μL (cDNA equivalent of 5 ng RNA) was used per ddPCR well.

The primers and probes used for ddPCR are shown in Table S2 and were purchased from Integrated DNA Technologies (Skokie, IL, USA). Assays were run in duplex (IFI6 on the FAM channel with MX1 on the HEX channel in one set of wells and ISG15 on the FAM channel with UBC on the HEX channel in a second set of wells). Each sample was run in duplicate for each assay. Sample pairs (i.e., on- and off-treatment) were always run on the same plates. ddPCR was performed using ddPCR Supermix for Probes (no dUTP), with droplets generated on a QX200 Automated Droplet Generator and droplets read on a QX200 Droplet Reader according to the manufacturer’s instructions (Bio-Rad, Hercules, CA, USA).

#### RNA sequencing in MTN-017

Total RNA prepared above was normalized to 300 ng input for library preparation with the TruSeq Stranded Total RNA with Ribo-Zero Globin kit (Illumina). The resulting libraries were assessed on the Agilent Fragment Analyzer with the HS NGS assay (Agilent) and quantified using the KAPA Library Quantification Kit (Roche) on a ViiA 7 Real Time PCR platform (Thermo Fisher). High depth sequencing (50 million reads per sample) was performed with a HiSeq 2500 (Illumina) on two High Output v4 flow cells as a 50 base pair, paired-end run.

#### Proteomics in MTN-017

Frozen rectal biopsies from MTN-017 were processed as described previously.^40^ For protein extraction, tissues were washed 3 times with 10 mM Tris (pH 7.6), placed in 5 mL of a lysis solution consisting of 7 M Urea, 2 M Thiourea, 40 mM Tris, and 10 mM DTT, and homogenized with a gentleMACS Octo Dissociator (RNA02-01M setting, Miltenyi Biotec, Bergisch Gladbach, Germany). Precipitates were removed by centrifugation at 9000 g for 20 minutes at 4°C, transfer of supernatant to a new tube, and a second round of centrifugation at 15,000 g for 20 minutes. Supernatants were stored at −80°C. Trypsin digestion was performed as described previously.^41^ Briefly, for each sample, 600 μL of tissue lysate was denatured in urea exchange buffer (8 M Urea in 1:10 0.5 M HEPES:water solution, GE HealthCare, Uppsala, Sweden) and filtered through a 10 kDa membrane. Filtered lysates were alkylated with 50 mM iodoacetamide for 20 minutes, and then washed with 50 mM HEPES buffer. Nucleic acids were removed by treatment with benzonase (150 units/μL in HEPES with MgCl_2_, Novagen, Darmstadt, Germany) for 30 minutes, and then lysates were washed with HEPES buffer. Trypsin digestion (2 μg trypsin per 100 μg protein, Promega, WI, USA) was performed overnight at 37°C. Eluted peptides were dried using a speed vacuum and then stored at −80°C. Reverse-phase liquid chromatography using a stepwise gradient was used to remove salts and detergents. Peptide quantification was performed with the LavaPep Fluorescent Protein and Peptide Quantification Kit (Gel Company, San Francisco, CA, USA). Mass spectrometry was performed using a nano flow liquid chromatography system (Easy nLC, Thermo Fisher) connected inline to a Velos Orbitrap mass spectrometer as described previously.^41^^,^^42^ One μg of peptide was run for each sample.

#### Immunofluorescence microscopy

Pairs of rectal and duodenal biopsies from eight subjects were stained for ISG15 protein for immunofluorescence microscopy. Duodenal biopsies from three subjects were also stained for glycoprotein 2 to identify M cells.^18^ Each pair consisted of one pre- and one on-treatment (∼60 days) sample from the ACTU-3500 study. Two rectal biopsies were of poor quality, so they and their pairs were excluded from analysis, reducing the sample size for the rectal biopsies to six pairs. The biopsies were collected into RNAlater Stabilization Solution (ThermoFisher), held at 4°C overnight, and then stored at −80°C. Prior to use, biopsies were thawed, fixed in 10% neutral buffered formalin for 3 days, and stored in 70% ethanol until paraffin embedding. Four micron thick sections were cut, attached to positively-charged slides and baked at 60°C for 1 h, with each slide holding one pre- and one on-treatment tissue section from the same participant. The histopathologist and data analyst were blinded to treatment status. Staining was performed using the procedure described previously.^43^ Primary antibodies were anti-ISG15 (Atlas Antibodies Cat#HPA004627, RRID: AB_1079152) and anti-glycoprotein 2 (GP2; Thermo Fisher, RRID: AB_2608499).

Slides were scanned on an Aperio FL (Version 2; Leica Biosystems). Exposure times were 125 ms for ISG15 and 64 ms for DAPI. Images were analyzed with HALO v2.2 image analysis software (Indica Labs, Albuquerque, NM, USA) with the CytoNuclear FL v1.4 algorithm. Images were annotated manually to select stroma or epithelium. Individual cells were identified by the software via DAPI-stained nuclei in conjunction with cell-defining parameters including nuclear contrast threshold, minimum nuclear intensity, nuclear segmentation aggressiveness, nuclear size, minimum nuclear roundness and maximum nuclear radius. These parameters were set to be optimal for each pair (i.e., settings were the same for the on- and off-treatment pairs for each person and sample type).

### Quantification and Statistical Analysis

Data were initially processed using instrument-specific software as described below. Following export from instrument-specific software, data were analyzed using R version 3.5.2. The following R packages from CRAN or Bioconductor^44^ were used: AnnotationDbi, Biobase,^44^ broom, conflicted, edgeR,^45^ ggrepel, here, limma,^39^ lumi,^38^ msigdbr, org.Hs.eg.db, pander, patchwork, plater,^46^ RColorBrewer, and tidyverse. R was run through RStudio version 1.1.463.

Statistical details of experiments can be found in this section and in the Results section. Sample sizes can be found in Table 1. Complete results of all analyses can be found in Data S1. Significance was defined as p < 0.05, following appropriate adjustment for multiple comparisons as described in the sections below. All code necessary to reproduce the analyses and figures is provided in Data S2.

#### Microarray analysis

Microarray analysis was done using R. All microarrays were pre-processed within study and sample type using variance stabilizing transformation^47^ and robust spline normalization from the lumi package.^38^ Probes that were rarely expressed in a given study arm were removed.

Differential gene expression was assessed using the limma package,^39^ which fits a linear model to each probe measured in the microarray, calculates empirical Bayes moderated t-statistics, and adjusts probe-level p values for multiple comparisons to control the false discovery rate using the method of Benjamini and Hochberg.^48^ In general, paired models were fit, with modeling done separately for each sample type and study (due to the separate preprocessing of different sample types). For the ACTU-3500 and MTN-017 study, within-participant comparisons were done comparing baseline samples to those obtained at the end of two months of treatment. Similar within-participant comparisons were done for the GMS A study, with the difference that samples during treatment were compared to samples taken two months after the end of treatment. For the GMS B study, treatment samples were compared to samples taken at the same time point from placebo recipients. Individual probes with an adjusted p value of less than 0.05 were defined as differentially expressed. Because there are multiple probes for some genes, probes were collapsed into genes by taking the probe with the lowest adjusted p value for each gene.^49^ Gene set testing was done using the camera function from limma,^50^ using the default inter-gene correlation of 0.01. The camera function is a competitive gene set, meaning that it tests whether the genes in a set are highly ranked as compared to genes outside of the set. The same threshold of 0.05 was used for gene sets, where p values were adjusted using the false discovery rate for the number of gene sets times the number of study arms tested (e.g., 50 Hallmark gene sets ∗ 11 study arms).

Thirteen cervical samples from the GMS A study clustered separately on principal components analysis plots from the rest of the cervical samples. Differential gene expression analysis comparing this cluster to the rest of the cervical samples revealed tens of thousands of genes to be differentially expressed and suggested that these samples may have included endocervical tissue, rather than only ectocervical as intended, possibly due to cervical ectopy. Keratin genes and gene ontology processes related to keratinocyte and epidermal development were higher in the main group of cervical samples, while the small cluster had higher expression of processes related to cilia movement and development, consistent with the ciliated epithelial cells of the endocervix. Because gene expression differed so dramatically in the thirteen cervical samples in question, we removed them from the analysis.

#### ddPCR analysis

The ddPCR data was analyzed using QuantaSoft version 1.7.4.0917 (Bio-Rad). The same fluorescence thresholds were applied to all samples across all plates. Wells with fewer than 10,000 droplets were removed. Concentrations of IFI6, MX1 and ISG15 were divided by the concentration of UBC from the corresponding sample to yield copies of each gene per copy of UBC. This value was log2-transformed to convert it to a normal distribution and place it on a comparable scale to the microarray data. Replicate wells were then averaged. Fold changes were calculated by subtracting the expression level from the off-treatment sample from the on-treatment sample.

#### RNA sequencing analysis

Raw demultiplexed fastq paired end read files were trimmed of adapters and filtered using the program skewer^51^ to remove any reads with an average phred quality score of less than 30 or a length of less than 36 bp. Trimmed reads were aligned using the HISAT2^52^ aligner to the *Homo sapiens* NCBI reference genome assembly version GRCh38 and sorted using SAMtools.^53^ Aligned reads were counted and assigned to gene meta-features using the program featureCounts^54^ as part of the Subread package. Counts data were analyzed analogously to the microarray data, using the voom function from limma and then fitting models for each transcript. Because the samples were processed in two batches, batch number was included in the model in addition to participant ID and treatment.

#### Mass spectrometry analysis

Feature detection, normalization, and quantification were performed using Progenesis LC-Mass Spectrometry software (Nonlinear Dynamics, Newcastle upon Tyne, UK) with default settings. Peptides were found using Mascot v.2.4.0 (Matrix Science, Boston, MA, USA) to search against the SwissProt database^55^ restricting taxonomy to Human. Search results were imported into Scaffold (Proteome Software, Portland, OR, USA) for peptide identification, requiring ≤ 0.1 FDR for protein identification, ≤ 0.01 FDR for peptide identification, and at least 2 unique peptides identified per protein. Samples were run in two batches, with US participants in one batch and Thai participants in the other. Data from the two cohorts were combined using Combat.^56^ Protein abundances were compared pairwise within participant using limma as described for microarray analysis.

#### Microscopy analysis

ISG15 intensity and the number of cells positive for ISG15 were compared by one-sided paired t test. The ISG15 signal was so bright that the software identified surrounding cells as positive for ISG15, despite manual inspection clearly showing that only one central cell was positive. To correct for this, hierarchical clustering was used on the spatial positions of the cells identified as positive. A distance threshold was empirically determined to count adjacent cells as a single positive cell, while still identifying nearby but distinct positive cells as distinct. The output of this analysis was verified by comparison to manual counting. Moreover, there was a strong correlation between the percentage of cells that were bright for ISG15 before and after adjustment for falsely positive surrounding cells (r = 0.98 for duodenum and 0.95 for rectum), the numbers were simply lower (and more reflective of manual inspection) after adjustment.

### Additional Resources

ACTU-3500 clinical trial: https://clinicaltrials.gov/ct2/show/NCT02621242

MTN-017 clinical trial: https://clinicaltrials.gov/ct2/show/NCT01687218

Partners PrEP clinical trial: https://clinicaltrials.gov/ct2/show/NCT00557245

## Supplemental Information

Document S1. Tables S1 and S2

Data S1. Results of Data Analysis, Related to Figures 1–4

Data S2. R Code Used for Data Analysis, Related to STAR Methods

Document S2. Article plus Supplemental Information
