# Supplementary material for: Treatment with Commonly Used Antiretroviral Drugs Induces a Type I/III Interferon Signature in the Gut in the Absence of HIV Infection
Source: Cell Rep Med. 2020 Sep 22;1(6):100096. doi: 10.1016/j.xcrm.2020.100096 (PMC7511692; doi:10.1016/j.xcrm.2020.100096)
Supplement: Document S1. Tables S1 and S2 [file mmc1.pdf]

Cell Reports Medicine, Volume *1*

Supplemental Information

Treatment with Commonly Used Antiretroviral Drugs Induces a Type I/III Interferon Signature in the Gut in the Absence of HIV Infection

Sean M. Hughes, Claire N. Levy, Fernanda L. Calienes, Joanne D. Stekler, Urvashi Pandey, Lucia Vojtech, Alicia R. Berard, Kenzie Birse, Laura Noël-Romas, Brian Richardson, Jackelyn B. Golden, Michael Cartwright, Ann C. Collier, Claire E. Stevens, Marcel E. Curlin, Timothy H. Holtz, Nelly Mugo, Elizabeth Irungu, Elly Katabira, Timothy Muwonge, Javier R. Lama, Jared M. Baeten, Adam Burgener, Jairam R. Lingappa, M. Juliana McElrath, Romel Mackelprang, Ian McGowan, Ross D. Cranston, Mark J. Cameron, and Florian Hladik

Supplemental Tables

Study	Sample	RIN
ACTU-3500	Duodenum	8.4 (7.8-9)
ACTU-3500	Whole blood	8.1 (7.1-8.7)
ACTU-3500	PBMC	9.6 (9.3-9.8)
ACTU-3500	Rectum	7.9 (6.9-8.8)
GMS A	Ectocervix	8.1 (7.1-8.8)
GMS A	PBMC	8.3 (6.5-9.4)
GMS A	Vagina	8.3 (6.5-9.7)
GMS B	PBMC	9.1 (7-9.7)
MTN-017	Rectum	7.8 (5.5-8.8)

Table S1. Sample quality. RNA Integrity Number (RIN) was determined by Agilent TapeStation. Values are displayed as mean (range). Related to Figures 1, 2, and 3.

Target	Assay Name	Component	Sequence
IFI6	Hs.PT.58.4407609	Probe	/56-FAM/CCA AGG TCT /ZEN/AGT GAC GGA GCC C/3IABkFQ/
		Primer 1	GTA GCA CAA GAA AAG CGA TAC C
		Primer 2	CTG CTG TGC CCA TCT ATC AG
MX1	Hs.PT.58.38362411	Probe	/5HEX/CTT GGA ATG /ZEN/GTG GCT GGA TGG C/3IABkFQ/
		Primer 1	CAT TCA GTA ATA GAG GGT GGG A
		Primer 2	TGA AAT CTG GAG TGA AGA ACG C
ISG15	Hs.PT.58.39185901.g	Probe	/56-FAM/CAC CTG GAA /ZEN/TTC GTT GCC CGC /3IABkFQ/
		Primer 1	GCC TTC AGC TCT GAC ACC
		Primer 2	CGA ACT CAT CTT TGC CAG TAC A
UBC	Hs.PT.39a.22214853	Probe	/5HEX/TCG ATG GTG /ZEN/TCA CTG GGC TCA AC/3IABkFQ/
		Primer 1	CCT TAT CTT GGA TCT TTG CCT TG
		Primer 2	GAT TTG GGT CGC AGT TCT TG

Table S2. Primers and probes used for ddPCR. Related to STAR Methods.
